# AP-2α Induces Epigenetic Silencing of Tumor Suppressive Genes and Microsatellite Instability in Head and Neck Squamous Cell Carcinoma

**DOI:** 10.1371/journal.pone.0006931

**Published:** 2009-09-09

**Authors:** Kristi L. Bennett, Todd Romigh, Charis Eng

**Affiliations:** 1 Genomic Medicine Institute, Lerner Research Institute and Taussig Cancer Institute, Cleveland Clinic, Cleveland, Ohio, United States of America; 2 Department of Genetics and Case Comprehensive Cancer Center, Case Western Reserve University School of Medicine, Cleveland, Ohio, United States of America; National Institute on Aging (NIA), National Institutes of Health (NIH), United States of America

## Abstract

**Background:**

Activator protein 2 alpha (AP-2α) is involved in a variety of physiological processes. Increased AP-2α expression correlates with progression in various squamous cell carcinomas, and a recent publication found AP-2α to be overexpressed in ∼70% of Head and Neck Squamous Cell Carcinoma (HNSCC) patient samples. It was found to repress transcription of the tumor suppressor gene C/CAAT Enhancer Binding Protein alpha (*C/EBPα*), and its binding site correlated with upstream methylation of the *C/EBPα* promoter. Therefore, we investigated the potential for AP-2α to target methylation to additional genes that would be relevant to HNSCC pathogenesis.

**Principal Findings:**

Stable downregulation of AP-2α stable by shRNA in HNSCC cell lines correlated with decreased methylation of its target genes' regulatory regions. Furthermore, methylation of *MLH1* in HNSCC with and without AP-2α downregulation revealed a correlation with microsatellite instability (MSI). ChIP analysis was used to confirm binding of AP-2α and HDAC1/2 to the targets. The effects of HDAC inhibition was assessed using Trichostatin A in a HNSCC cell line, which revealed that AP-2α targets methylation through HDAC recruitment.

**Conclusions:**

These findings are significant because they suggest AP-2α plays a role not only in epigenetic silencing, but also in genomic instability. This intensifies the potential level of regulation AP-2α has through transcriptional regulation. Furthermore, these findings have the potential to revolutionize the field of HNSCC therapy, and more generally the field of epigenetic therapy, by targeting a single gene that is involved in the malignant transformation via disrupting DNA repair and cell cycle control.

## Introduction

The Activator Protein-2 family is composed of five members (α, β, γ, ε, δ). These genes are transcription factors that play roles in apoptosis, cell cycle regulation, tissue differentiation, and development [1]. Activator protein 2 alpha (AP-2α) is involved in a variety of processes, including adipogenesis [2], neuronal development [1], and is suspect in cancer progression [3]. AP-2α binds as a homo or heterodimer to targets that contain the motif “GCCNNNGGC” in their regulatory regions [4]. After binding, AP-2α can either function as a transcriptional activator or repressor [5], [6]. It is normally expressed at very low levels in keratinocytes, and it is elevated in proliferating, malignant keratinocytes of squamous cell carcinoma of the skin [7]. Furthermore, UVA irradiation increases AP-2α expression and results in malignant transformation [3], [8]. Because AP-2α transcriptionally regulates such genes as *p21* and *CDH1*, it is feasible to propose its relevance in tumorigenesis [9]. In fact, AP-2α has been shown to act as both a tumor suppressor and an oncogene in different cancer types [3], [5], [8]. It appears this is determined by the type and quantity of AP-2 isoforms and AP-2 modulating factors present in the cell [9].

Histone and promoter DNA methylation have been revealed in the past decade to be a frequent cause of gene silencing of tumor suppressors in a variety of malignancies [10], [11]. DNA methylation sterically inhibits transcription factor binding, thereby inhibiting gene activation [12]. Conversely, decreased gene transcription has been shown in some cases to precede and propogate DNA methylation, resulting in more stable gene downregulation. For example, a transcriptional suppressor protein that competes with a transcriptional activator for the same binding site could result in decreased activation through increased methylation [13].

AP-2α is one such example, providing transcriptional repression to some genes which are also epigenetically modified[14], [15]. It competes with the activator SP1 for binding to these target promoters, thereby repressing transcription [2], [16]. AP-2α has previously been shown to recruit HDACs to specific gene promoters following retinoic acid induction [15], and a recent study observed that regions of frequent methylation in Acute Lymphoblastic Leukemia often contained AP-2α binding sites [17]. Therefore, it is reasonable to hypothesize that in cancer cells AP-2α may bind to specific target genes, silence transcription, and propagate DNA methylation in order to stably silence tumor suppressors and DNA repair genes.

The following study investigates 8 target genes in HNSCC for the possibility of AP-2α-induced targeted methylation and subsequent gene silencing. Decreased AP-2α expression was found to correlate with decreased target methylation, increased target gene expression, and decreased microsatellite instability. ChIP analysis revealed an association between AP-2α and HDAC binding to target promoters. Also, the effect of HDAC inhibition on target methylation mirrored that of AP-2α downregulation. Therefore, the mechanism of AP-2α-induced methylation is likely explained by HDAC recruitment.

## Results

### AP-2α downregulation decreases target gene methylation

**Figure 1:**
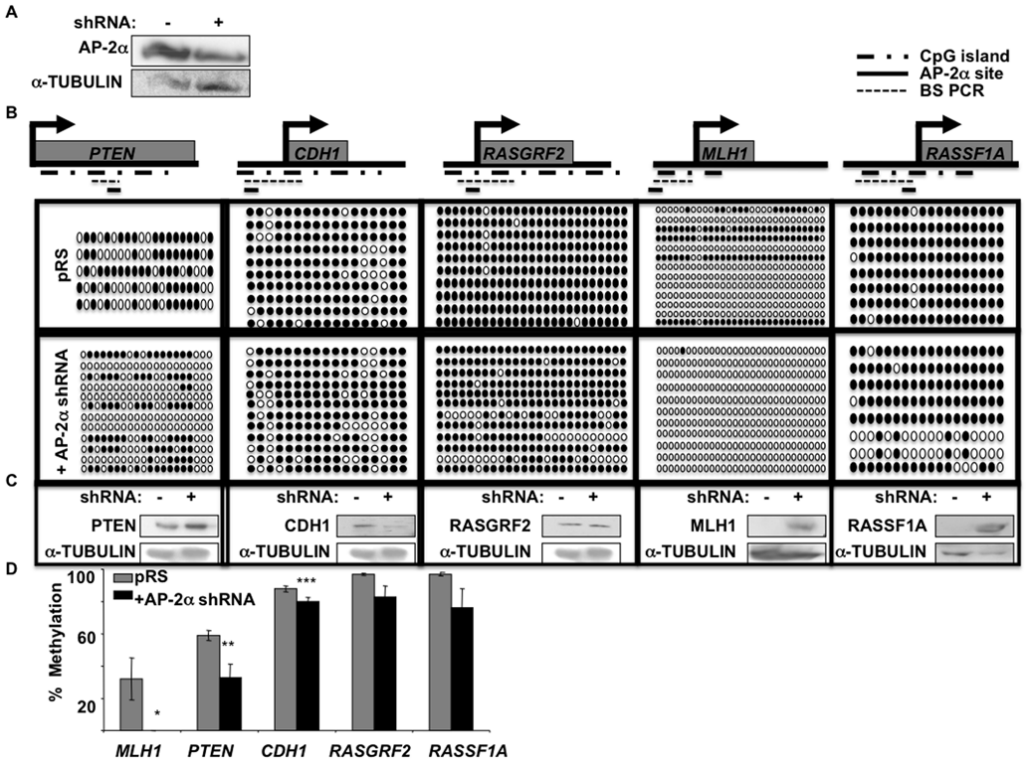
Targeted methylation and repression via AP-2α in HNSCC cell lines SCC22B. a) Western blot analysis to confirm AP-2α downregulation in SCC22B. b) Via bisulfite sequencing analysis, methylation of 5 target genes was compared between SCC22B cells with and without AP-2α expression. Gene drawings indicate the CpG island and bisulfite PCR product in relationship to the transcription start site (arrow). Solid circles represent methylated CpGs; whereas, open circles represent unmethylated CpG sites. The small bars represent the location of the predicted AP-2α site. c) Western blot analysis was performed to analyze methylation's effect on target expression. d) Graphical representation of the average methylation percentage in the 5 target genes analyzed. * P-value = 0.042; ** P-value = 0.040; *** P-value = 0.014.

Using a previously generated HNSCC cell line (SCC22B) in which AP-2α is stably downregulated [14](Figure 1a), DNA methylation was analyzed in 16 potential AP-2α target genes' promoters (list shown in Table S1). These genes were selected based on previous evidence for methylation in a variety of malignancies and potential regulation by AP-2α (i.e. the presence of consensus AP-2α binding sites in the target's promoter). A preliminary methylation screen was performed in wildtype SCC22B cells, which yielded methylation in 8/16 of the targets tested: *RARB2*, *DCC*, *DAPK*, *PTEN*, *MLH1, CDH1*, *P73*, and *RASSF1A* (Figure 1b and Figure S2). Bisulfite sequencing was then performed on these eight genes in SCC22B cells with and without AP-2α downregulation (detailed gene diagrams are displayed in Figure S1). Of these eight genes, five genes (*RARB2, PTEN*, *MLH1*, *CDH1*, and *RASSF1A*) showed some level of demethylation in the SCC22B cells with AP-2α downregulation compared to the wildtype cells (Figure 1b; graphical representation in Figure 1d).

### Decreased target gene methylation correlates with decreased expression

In order to assess the significance of the target methylation, western blot analysis was performed on lysates from the wildtype and AP-2α-downregulated SCC22B cells. This revealed a visible increase in protein expression in 3/5 of the genes (i.e. *PTEN*, *MLH1*, and *RASSF1A*) and no detectable change in *RASGRF2* (Figure 1c). Not surprisingly, *CDH1* showed a decrease in protein expression in response to AP-2α-downregulation, which correlates with previous findings that AP-2α is required for activation, not repression, of this gene (Figure 1c)[18]. AP-2α has previously been shown to regulate *PTEN* [19], but the findings of AP-2α regulation of *MLH1* and *RASSF1A* are novel.

**Figure 2:**
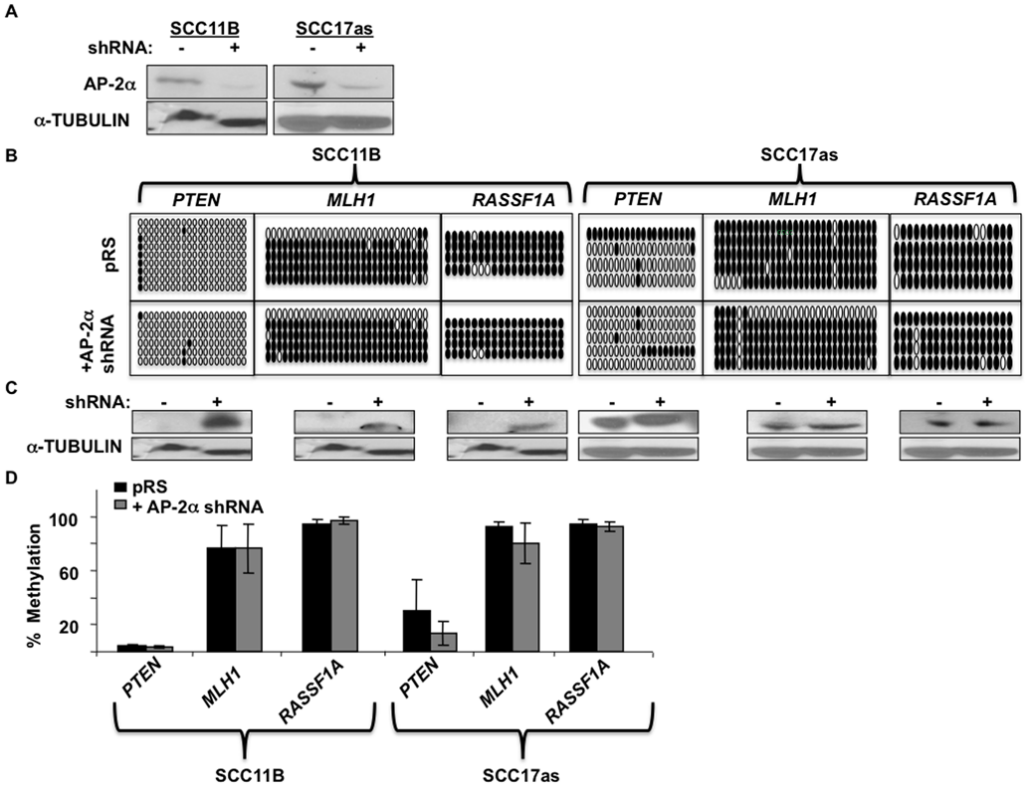
Varied target methylation and expression following AP-2α downregulation in HNSCC cell lines SCC11B and SCC17as. a) Western blot analysis to confirm AP-2α downregulation in SCC11B and SCC17as. b) Methylation in 3 target genes was compared between SCC11B and SCC17as cells with and without AP-2α expression. Solid circles represent methylated CpGs; whereas, open circles represent unmethylated CpG sites. c) Western blot analysis was performed to analyze methylation's affect on target expression. d) Graphical representation of the average methylation percentage in the 3 target genes analyzed.

AP-2α was also downregulated in additional HNSCC cell lines (SCC11B and SCC17as)(Figure 2a). The methylation status of the 3 genes with decreased methylation and increased expression in AP-2α-downregulated SCC22B cells (i.e. *RASSF1A*, *MLH1*, and *PTEN*) was analyzed. SCC17as displayed subtle decreases in *PTEN* and *MLH1* methylation in the AP-2α-downregulated cells (Figure 2b and 2d), and both SCC11B and SCC17as revealed increased target gene expression after AP-2α downregulation (Figure 2c). AP-2α-targeted methylation was also investigated in malignancies other than HNSCC, and colon cancer was selected based on the association of *MLH1* methylation [20], [21], [22]. AP-2α was downregulated in two colon cancer cell lines, HCT116 and HT29 (Figure S3a). The region analyzed for MLH1 showed insignificant methylation levels in the vector-only cancer cells for both HT29 and HCT116 (Figure S3b). However, both *PTEN* and *RASSF1A* demonstrated slightly higher levels of methylation, which were decreased in correlation with AP-2α downregulation in both HT29 and HCT116 (Figure S3c).

### AP-2α downregulation seems to be associated with decreased MSI

**Figure 3:**
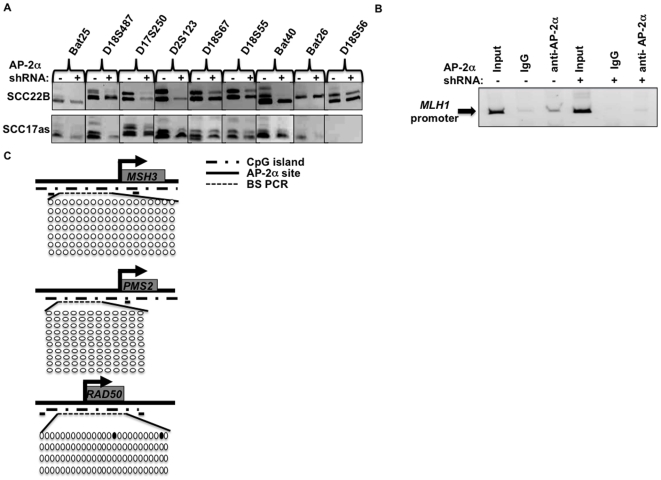
Decreased MSI following AP-2α downregulation in HNSCC cell lines SCC22B and SCC17as. a) 9 MSI markers were used with DNA from SCC22B and SCC17as cells with and without AP-2α downregulation, and the products were analyzed on an 8% PAGE gel. b) ChIP analysis using an AP-2α antibody in SCC22B cells with and without AP-2α downregulation. ChIP eluate was then utilized for quantitative PCR using MLH1 promoter primers. c) Bisulfite sequencing analysis of 3 additional DNA repair genes in WT SCC22B cells. Because no methylation was observed in the WT cells, it was unnecessary to analyze methylation in AP-2α downregulated cells. Solid circles represent methylated CpGs; whereas, open circles represent unmethylated CpG sites.

Methylation of *MLH1*, a DNA repair gene, has been previously shown to correlate with microsatellite instability in colon, gastric, and endometiral cancers [20], [21], [22]. Therefore, MSI was compared in SCC22B, SCC17as, SCC11B, HCT116, and HT29 cells with and without AP-2α downregulation. Interestingly, MSI primers yielded fewer products in both SCC22B and SCC17as AP-2α-downregulated cells compared to the control cells (Figure 3a) (SCC22B = Bat25, D18S487, D17S250, D2S123, Bat40, and D18S56; SCC17as = Bat25, D18S487, D2S123, D18S67, and Bat40; D18S56 did not amplify in SCC17as). This suggests a potential decrease in MSI in these cells correlating with decreased AP-2α (Figure 3a). As expected, the cell lines that exhibited no change in MLH1 methylation (SCC11B, HT29, and HCT116) in the region tested did not reflect any changes in MSI with AP-2α downregulation (data not shown).

In order to confirm AP-2α actually binds to the *MLH1* promoter, ChIP analysis was performed in SCC22B with and without AP-2α downregulation. This revealed AP-2α does bind to the *MLH1* promoter, and this binding is decreased in the AP-2α-downregulated cells (Figure 3b). Methylation screening was performed for additional DNA repair genes (i.e. *MSH3, PMS2*, and *RAD50*) that contained both CpG islands and potential AP-2α binding sites within their promoter elements. This bisulfite sequencing analysis revealed relatively no methylation of these genes (Figure 3c).

### Decreased AP-2α and HDAC1/2 binding to target promoters following AP-2α downregulation

**Figure 4:**
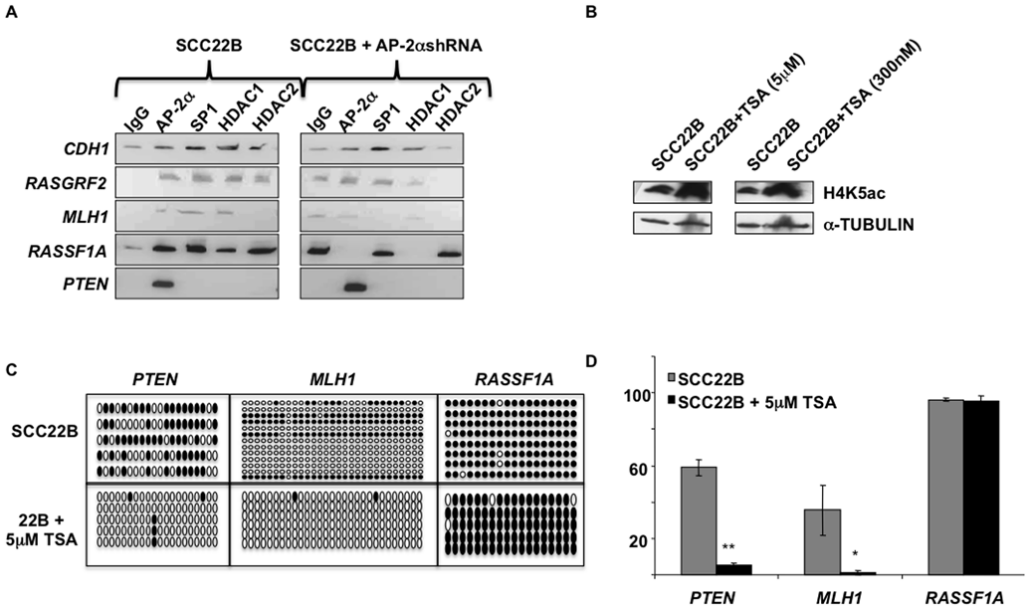
AP-2α interacts with HDAC1/2, and HDAC activity is required for targeted methylation in HNSCC cells. a) ChIP analysis was performed using AP-2α, SP1, HDAC1, and HDAC2 antibodies in SCC22B cells with and without AP-2α downregulation. ChIP eluate was then utilized for quantitative PCR using *CDH1*, *RASGRF2*, *MLH1*, *RASSF1A*, and *PTEN* promoter primers. b) Western blot analysis demonstrating increased acetylation following Trichostatin A (TSA) treatment. c) Bisulfite sequencing analysis was performed in SCC22B cells before and after TSA treatment. Solid circles represent methylated CpGs; whereas, open circles represent unmethylated CpG sites. d) Graphical representation of the average methylation percentage in the 3 target genes analyzed. * P-value = 0.0452; ** P-value = 3.77×10^−6^.

In a previous publication, AP-2α binding to target promoters was shown to precede recruitment of HDAC proteins to the region for more stable silencing [15]. Therefore, to address the mechanism by which AP-2α may affect gene methylation and silencing in these HNSCC cells, ChIP analysis was performed on SCC22B cells with and without downregulation of AP-2α. As expected, this semi-quantitative analysis revealed AP-2α and HDAC1/2 binding to all of the target promoters tested, except *PTEN*, in the wildtype SCC22B cells (Figure 4a). Furthermore, both AP-2α and HDAC binding was reduced in the AP-2α downregulated cells. HDAC1 was found to be more frequently associated with the targets compared to HDAC2, which did not pulldown *RASSF1A*. Since AP-2α and SP1 bind to the same consensus sequence, SP1 binding was also investigated in order to assess whether increased target gene expression was due to the restored binding of the activator SP1 following removal of AP-2α. With the exception of *CDH1*, decreased AP-2α binding did not allow increased SP1 binding as would have been expected.

### HDAC inhibition's effect on target methylation correlates with AP-2α downregulation

In order to investigate whether the mechanism of AP-2α-targeted methylation involved HDAC recruitment and activity, class I and II HDACs were inhibited via Trichostatin A (TSA). Efficacy of TSA treatment was verified via western analysis for acetyl histone H4K5 (Figure 4b). Following TSA treatment, target methylation was then compared with that of the SCC22B cells with intact HDAC activity. Both *PTEN* and *MLH1* demonstrated a significant decrease in methylation subsequent to TSA treatment; however, *RASSF1A* remained unchanged (Figure 4c; ** P-value = 3.77×10^−6^ and * 0.0452, respectively; graphical quantitation shown in Figure 4d). This correlates with previous findings that TSA alone is insufficient to change the histone acetylation status of *RASSF1A* [23]. However, no obvious change in target gene expression resulted from the increased acetylation (data not shown). This may be due to the brief time course (24 hours), which may not allow enough time for the methylation changes to reflect their impact on expression [24].

### AP-2α downregulation correlates with decreased mitosis

**Figure 5:**
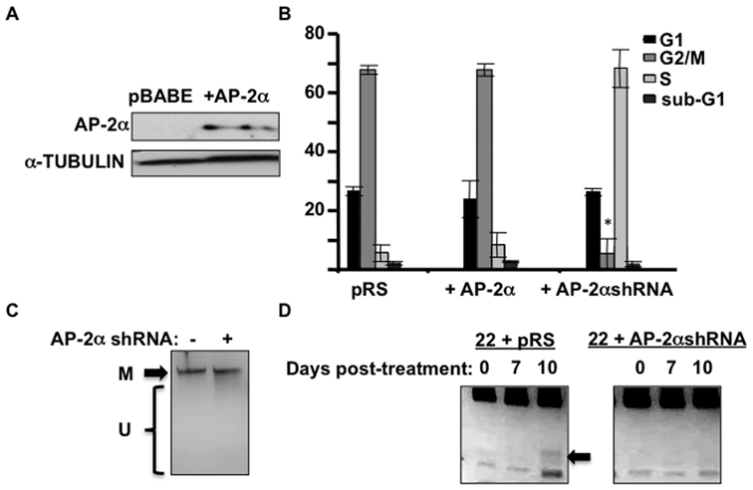
Decreased AP-2α expression reduces proliferation; and AP-2α is required for target remethylation following demethylation. a) AP-2α western blot analysis of SCC22B cells with AP-2α overexpression. b) Cell cycle analysis via flow cytometry comparing WT, AP-2α-overexpressing, and AP-2α-downregulated SCC22B cells. * P-value = 0.0003. c) Methylation sensitive HhaI digests were performed on DNAs from SCC22B cells with and without AP-2α downregulation, revealing no distinguishable difference in global DNA methylation. “M” = methylated; “U” = unmethylated. d) 1 µM 5-aza-2′deoxycytidine was applied to the cells for 96 hours, changing the media daily. After 96 hours, the 5-aza-2′deoxycytidine was removed and the cells were continued in culture for 10 days, collecting cells at 0, 7, and 10 days post-treatment. Combined bisulfite restriction analysis on bisulfite-treated DNA obtained from these cells at 0, 7, and 10 days. Increased methylation at 10 days is seen in the AP-2α-expressing cells (i.e. the digested band indicated by the arrow).

Because AP-2α appears to target methylation and silencing of certain tumor suppressor genes in this HNSCC cell line, downregulation of AP-2α (i.e. relief of tumor suppressor silencing) should correlate with decreased proliferation. We performed cell cycle analysis by flow cytometry on the HNSCC cells SCC22B with endogenous AP-2α, AP-2α overexpression (Figure 5a), and downregulation of AP-2α. The pattern in AP-2α overexpressing cells looked similar to endogenous AP-2α expression; however, HNSCC cells with AP-2α downregulation exhibited significantly less cells in mitosis (Figure 5b; P-value = 0.0003).

### Lack of AP-2α inhibits remethylation of *PTEN* following 5-aza-2′ deoxycytidine treatment

In order to exclude the possibility that AP-2α is affecting DNA methylation at the global level, an methylation sensitive digest was performed on DNAs from SCC22B cells with and without AP-2α downregulation. This revealed no substantial difference in DNA methylation levels between the two cell types (Figure 5c), suggesting the DNA methylation changes from AP-2α are gene-specific. To determine whether AP-2α acts to maintain or induce this specific target DNA methylation, the HNSCC cells SCC22B (+/− AP-2α shRNA) were treated with a demethylating drug (5-aza-2′deoxycytidine) and then permitted to recover methylation by continued culturing in the absence of the drug. Cells collected at 7 days post-treatment showed no *PTEN* methylation in both cell types (Figure 5d). However, after 10 days *PTEN* methylation was returning in the SCC22B cells with AP-2α expression, whereas, *PTEN* remained unmethylated in the cells lacking AP-2α expression (Figure 5d). Therefore, this is suggestive of a mechanism by which AP-2α induces target methylation.

## Discussion

It has previously been shown that AP-2α is expressed at relatively low levels in normal keratinocytes and is significantly elevated in cancerous and proliferating keratinocytes in squamous cell carcinoma of the skin [7]. Forced expression of AP-2α in keratinocytes causes increased expression of the oncogene Amyloid Precursor Protein (APP), and these two genes have both been found to be increased in squamous cell carcinomas [25]. Furthermore, AP-2α interacts with *SMAD2/3* to affect TGFΒ signaling, which is frequently disrupted in HNSCC [26]. These findings reveal the potential importance of AP-2α's transcriptional regulation of target genes in the pathogenesis of HNSCC.

It has been suggested that AP-2α's ability to repress target genes results in cellular transformation and tumorigenesis [27], and decreased transcriptional suppression of AP-2α targets via abolished target binding has been shown to favor cellular differentiation [2], [28]. In agreement, the results of this study in HNSCC reveal that downregulating AP-2α increases target gene expression by decreasing promoter DNA methylation and results in decreased cellular proliferation. In addition to its effects on proliferation, AP-2α also has been shown to potentially impact microsatellite instability through its interaction with p53 and subsequent *RAD51* suppression [29]. In this study, we demonstrate that AP-2α expression correlates with increased MSI in HNSCC cell lines, which correlates with *MLH1* methylation and decreased expression.

It has been demonstrated that AP-2α may help to recruit HDACs to the promoters to which it is bound [15]. Therefore, we investigated the association between AP-2α and HDAC binding to target promoters. This revealed a correlation between AP-2α and HDAC binding, and HDAC inhibition via TSA yielded similar effects on target demethylation as AP-2α downregulation. In fact, in some cases such as SCC11B and SCC17as, deacetylation via HDAC recruitment may be the primary means of target silencing by AP-2α (since AP-2α downregulation yielded no accountable methylation changes). Therefore, this study reveals that AP-2α acts as a suppressor for certain “tumor suppressive” genes in HNSCC by targeting promoter methylation and/or deacetylation via HDAC recruitment.

Perhaps most interesting is the novel discovery that AP-2α is not only involved in the epigenetic silencing of tumor suppressor and DNA repair genes, but also appears to contribute to microsatellite instability in HNSCC. In this manner, AP-2α allows for both increased tumor proliferation and increased genomic instability. Furthermore, this phenomenon in HNSCC is distinct from the colon cancer cell lines used in this study, in which MSI appears to be unaffected by AP-2α expression changes. In light of a recent publication demonstrating AP-2α overexpression in 69% of HNSCC patients [14], these findings emphasize the impact AP-2α has in HNSCC pathogenesis. Therefore, clinical treatment that targets AP-2α expression in the context of the MSI-positive HNSCC tumor would potentially deter tumor proliferation (ie. via *PTEN* and *RASSF1A* reexpression) and resolve genomic instability (ie. via *MLH1* reexpression).

## Methods

### Cell lines, antibodies, and plasmids

The established human HNSCC cell lines used in the study (SCC22B, SCC11B, and SCC17as) were kindly provided by Dr. Thomas Carey, University of Michigan. Cell lines were maintained in Dulbecco's Modified Eagle's Medium (DMEM) with 10% Fetal Bovine Serum (FBS) and 1% streptomycin/penicillin (S/P) antibiotics. MLH1 (abcam; ab9144), CDH1 (Cell Signaling; 4065), RASGRF1/2 (Santa Cruz; sc-28580), RASSF1A (abcam;ab23950), α-TUBULIN (Cell Signaling Technologies; Danvers, MA), PTEN (monoclonal antibody clone 6H2.1; Cascade Biosciences, Portland, OR), AP-2α (abcam; ab18112), and acetyl Histone H4 Lys 5 (Upstate Cell Signaling Solutions; 07–327). The AP-2α shRNA construct was previously described [14]. The AP-2α overexpression construct was made by PCR amplification of the complete AP-2α cDNA sequence, using primers with EcoRV and SalI ends. Blunt-end ligation was performed using pBABE following BamHI digestion and Klenow polymerase reaction.

### DNA isolation

DNA was isolated using the DNA QiaAmp kit (Qiagen) according to the manufacturer's protocol.

### Trichostatin A (TSA) treatment

The SCC22B cell line was treated with either 300 nM or 5 µM TSA for 24 hours. The cells were then collected for western blot analysis and DNA isolation. The 5 µM treated cells were used for the bisulfite sequencing analysis.

### 5-aza-2′deoxycytidine treatment

Cells were treated with 1 µM of the drug (Sigma-Aldrich) for 96 hours while maintaining ∼50% confluency and changing the drug daily.

### Western blot analysis

Whole cell lysates were suspended in lamaelli buffer and boiled for 15 minutes, followed by centrifugation. Lysates were loaded onto a 7.5% acrylamide gel and run for 2 hours at 110 volts followed by semidry transfer of the proteins to nitrocellulose membranes. The membranes were then blocked in 5% milk for 1 hour. 1∶1,000 dilution of the primary antibodies were incubated with the blot overnight shaking at 4 degrees Celsius. A one hour incubation with the secondary antibodies (1∶5,000 dilution) followed. The blots were then washed in 1x TBS-Tween for 1 hour and films exposed using ECL solutions. Western blots are representative of three independent experiments.

### MSI analysis

Commonly used MSI primers were utilized for the analysis (Bat25, Bat26, Bat40, D2S123, D17S250, D18S55, D18S56, D18S67, and D18S487)[30], [31], [32], [33]. DNA was isolated from HNSCC SCC22B cells with and without AP-2α downregulation using the Qiagen DNA mini kit according to the standard protocol (Qiagen). 5 ng of this DNA (1 µl) was used for PCR along with 10 µl HotStart mix (Qiagen), 6 µl water, and 2 µl primer (20 pmol). The PCR products were subsequently subjected to electrophoresis on an 8% polyacrylamide gel.

### Combined Bisulfite Restriction Analysis (COBRA)

COBRA was performed as previously described [34]. Primer sequences will be provided upon request.

### Bisulfite sequencing analysis

Bisulfite sequencing was performed as previously described [35]. Primer sequences will be provided upon request.

### Chromatin immunoprecipitation analysis

ChIP analysis was done as previously described [34], according to the Upstate Cell Signaling Solutions protocol. Sequences of the primers used for the quantitative ChIP PCRs can be provided upon request.

### Hha I digest

Global methylation status was analyzed in SCC22B cells +/− AP-2α shRNA. 1ug of DNA was incubated with 1 unit HhaI (New England Biolabs) at 37 degrees Celsius for 3 hours. The digests were then loaded onto a 1% agarose gel for comparison.

## Supporting Information

Table S1 (0.08 MB RTF)

Figure S1 Diagram of 5 genes displaying the amplified region in respect to the corresponding gene and associated CpG island. Bisulfite sequencing analysis was performed on five genes which yielded differences in methylation following AP-2α downregulation in HNSCC. The diagram shows the location of the bisulfite PCR product, the CpG island, and the gene layout. The numbers provided are in respect to the transcription start site.
(2.55 MB DOC)

Figure S2 Some methylated genes in HNSCC are unaffected by AP-2α downregulation. Bisulfite sequencing analysis was performed on three genes which yielded no substantial differences in methylation following AP-2α downregulation. Solid circles represent methylated CpGs; whereas, open circles represent unmethylated CpG sites.
(2.07 MB TIF)

Figure S3 AP-2α downregulation in colon cancer cell lines causes a subtle decrease in RASSF1A and PTEN methylation. a) Western blot analysis showing AP-2α downregulation in HT29 and HCT116 cell lines. b) Bisulfite sequencing analysis on MLH1 shows little methylation in WT HCT116 and HT29 cell lines. c) Bilsulfite sequencing analysis on RASSF1A and PTEN in HCT116 and HT29 colon cancer cell lines with and without AP-2α downregulation. Solid circles represent methylated CpGs; whereas, open circles represent unmethylated CpG sites.
(1.67 MB TIF)
