# Supplementary material for: AP-2α Induces Epigenetic Silencing of Tumor Suppressive Genes and Microsatellite Instability in Head and Neck Squamous Cell Carcinoma
Source: PLoS One. 2009 Sep 9;4(9):e6931. doi: 10.1371/journal.pone.0006931 (PMC2734430; doi:10.1371/journal.pone.0006931)
Supplement: Table S1 — (0.08 MB RTF) [file pone.0006931.s001.rtf]

Table S1. Selection of potential AP2α methylation targets. 
Genes with AP-2 binding sites	Head and Neck	Colon	Prostate	Breast	Brain	Lung	
CDKN2A	X	X	X	X	X	X	
PTEN	X, Y	X	X	X	X	X	
APC	X	X	X	X	X	X	
MLH1	X, Y	X	X	X	X	X	
DCC	X, Y	X		X		X	
RASSF1A	X, Y	X	X	X	X	X	
DAPK	X, Y	X	X	X	X	X	
MGMT	X	X	X	X	X	X	
RARB2	X	X	X			X	
SALL3		X	X		X	X	
P73	Y	X	X	X	X	X	
E-CADHERIN	X, Y	X	X	X	X	X	
RASGRF2	Y	X	X		X	X	
VIMENTIN		X		X	X	X	
SLC16A12		X	X	X			
DPYS		X	X	X			
Genes were selected from the literature based on frequent methylation in a variety of cancers (31 genes). Methylation analysis was performed on 16/31 of the selected candidates (which are displayed in the table), because they were found to contain a putative AP2α binding site in their regulatory sequence. X = genes found to be methylated from previous studies; Y= genes found to be methylated in the present study. Gene names that are underlined were found to be both methylated in HNSCC and changed by AP2α expression.
