# Supplementary figures and images for: AP-2α Induces Epigenetic Silencing of Tumor Suppressive Genes and Microsatellite Instability in Head and Neck Squamous Cell Carcinoma
Source: PLoS One. 2009 Sep 9;4(9):e6931. doi: 10.1371/journal.pone.0006931 (PMC2734430; doi:10.1371/journal.pone.0006931)

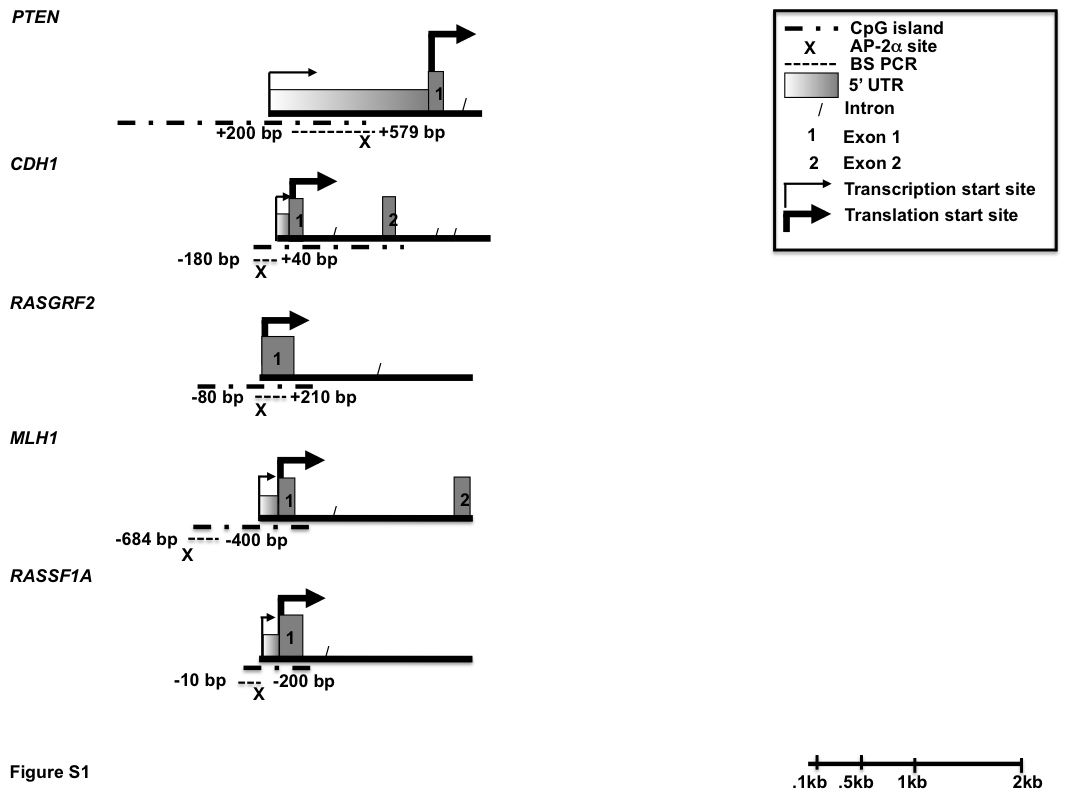

Supplement: Figure S1 — Diagram of 5 genes displaying the amplified region in respect to the corresponding gene and associated CpG island. Bisulfite sequencing analysis was performed on five genes which yielded differences in methylation following AP-2α downregulation in HNSCC. The diagram shows the location of the bisulfite PCR product, the CpG island, and the gene layout. The numbers provided are in respect to the transcription start site. (2.55 MB DOC) [file pone.0006931.s002.tif]

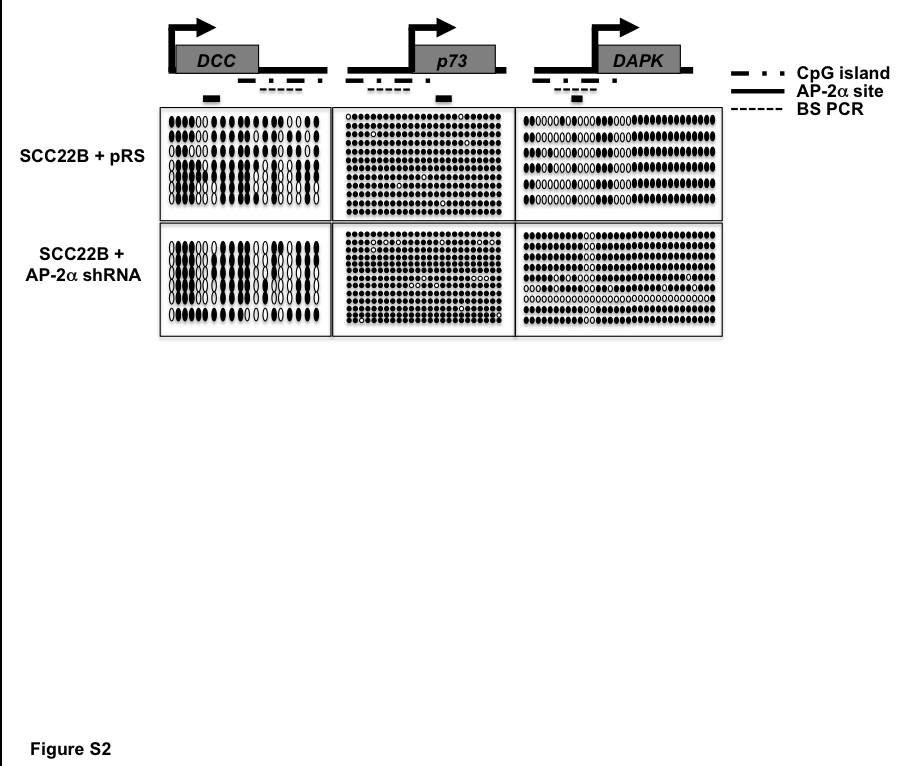

Supplement: Figure S2 — Some methylated genes in HNSCC are unaffected by AP-2α downregulation. Bisulfite sequencing analysis was performed on three genes which yielded no substantial differences in methylation following AP-2α downregulation. Solid circles represent methylated CpGs; whereas, open circles represent unmethylated CpG sites. (2.07 MB TIF) [file pone.0006931.s003.tif]

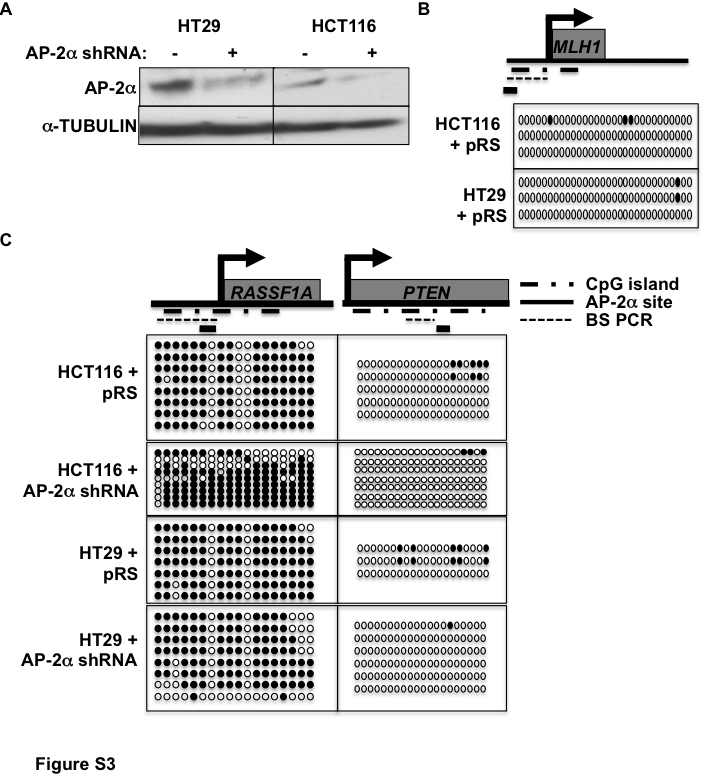

Supplement: Figure S3 — AP-2α downregulation in colon cancer cell lines causes a subtle decrease in RASSF1A and PTEN methylation. a) Western blot analysis showing AP-2α downregulation in HT29 and HCT116 cell lines. b) Bisulfite sequencing analysis on MLH1 shows little methylation in WT HCT116 and HT29 cell lines. c) Bilsulfite sequencing analysis on RASSF1A and PTEN in HCT116 and HT29 colon cancer cell lines with and without AP-2α downregulation. Solid circles represent methylated CpGs; whereas, open circles represent unmethylated CpG sites. (1.67 MB TIF) [file pone.0006931.s004.tif]
